# Predicting Lifetime Risk of Kidney Failure Using Age and a Single eGFR Measurement

**DOI:** 10.3390/jcm15072653

**Published:** 2026-03-31

**Authors:** Ryo Enoki, Mariko Miyazaki, Enyu Imai, Tetsuhiro Tanaka, Koji Okamoto

**Affiliations:** 1 Department of Nephrology, Graduate School of Medicine, Tohoku University, Sendai 980-8574, Japan; 2 Nakayamadera Imai Clinic, 2-8-18 Nakayamadera, Takarazuka 665-0861, Japan

**Keywords:** chronic kidney disease progression, age–eGFR index, single-point risk assessment, eGFR trajectory modeling

## Abstract

**Background:** The prognosis of chronic kidney disease (CKD) typically requires longitudinal estimated glomerular filtration rate (eGFR) data, making risk stratification difficult at initial consultation. Furthermore, eGFR-based clinical decisions often overlook the critical factor of patient age. This study aimed to establish a simplified predictive model for progressive CKD and quantify the impact of clinical interventions. **Methods:** Utilizing a historical dataset (1988–2003) from the pre-renin-angiotensin system inhibitor (RASi) and pre-sodium-glucose cotransporter 2 inhibitor (SGLT2i) era, we developed heatmaps to predict the probability of reaching eGFR < 30 mL/min/1.73 m^2^ by age 80 years. The model also estimated the risk reduction from smoking cessation and pharmacological therapies. The predictive performance for age + eGFR was assessed using standard calibration and discrimination metrics, and clinical utility was evaluated using decision curve analysis across a range of threshold probabilities. Risk reclassification analyses compared age +eGFR-based categories with conventional eGFR-based stratification. **Results:** Regarding the risk of eGFR < 30 mL/min/1.73 m^2^ by age 80 years, simulations confirmed a correlation between age and eGFR. At age 40 years, an eGFR of ~57 mL/min/1.73 m^2^ indicated a 50% probability of progressing to CKD stage 4 by age 80 years. This threshold decreases to 53 and 48 mL/min/1.73 m^2^ at 50 and 60 years of age, respectively. Calibration and discrimination analyses demonstrated acceptable agreement between predicted and observed risks. Decision curve analysis showed that an age + eGFR threshold of approximately 115 primarily provided a net benefit at lower threshold probabilities, supporting intensified surveillance strategies, whereas an age +eGFR of 100 showed a positive net benefit across a broader range of thresholds, comparable to the conventional eGFR < 45 mL/min/1.73 m^2^ criterion. While proteinuria markedly increased risk, smoking cessation provided a 9.4–11.2% risk reduction. Combined RASi and SGLT2i treatment showed the greatest impact, reducing progression probability by 31.2–40.0% (e.g., reducing a 50.0% baseline risk to 32.1% in 40-year-old men). **Conclusions:** The age + eGFR rule represents a simple, clinically interpretable heuristic for age-adjusted risk stratification based on a single eGFR measurement and may offer potential clinical utility for guiding surveillance intensity and consideration of earlier intervention strategies. However, external validation is required before clinical application.

## 1. Introduction

Chronic kidney disease (CKD) was defined as a clinical entity by the Kidney Disease Outcomes Quality Initiative (KDOQI) in 2002 [1,2] in response to the growing global burden of kidney dysfunction and the recognition that reduced kidney function and proteinuria are major risk factors for cardiovascular disease and mortality. CKD affects an estimated 700–800 million adults worldwide [1,2].

The current evaluation of CKD is based on the CGA classification, which includes disease cause (C), glomerular filtration rate (G), and albuminuria (A). In addition to these parameters, the estimated glomerular filtration rate (eGFR) decline, expressed as the eGFR slope, serves as an important indicator of CKD progression [3]. A steep eGFR slope over time is associated with a more rapid progression to progressive CKD, and a decline exceeding −5.0 mL/min/1.73 m^2^ per year is generally considered rapid progression [4]. Reported eGFR slopes vary, including −1.03 mL/min/1.73 m^2^ per year in a European CKD cohort [5] and −1.4 and −0.8 mL/min/1.73 m^2^ per year in older Canadian men and women without diabetes, respectively [6].

Renin-angiotensin system inhibitors (RASis), including angiotensin-converting enzyme inhibitors (ACEIs) and angiotensin II receptor blockers (ARBs), have been shown in multiple meta-analyses [7,8] and randomized controlled trials (IDNT [9] and RENAAL [10]) to reduce CKD progression and all-cause mortality across CKD stages, irrespective of diabetes status. These trials demonstrated that ARB therapy reduces the rate of eGFR decline by approximately 30%.

Recently, sodium-glucose cotransporter 2 inhibitors (SGLT2is) have emerged as a cornerstone of renoprotective therapy. Large-scale trials, including CREDENCE [11], DAPA-CKD [12], and EMPA-KIDNEY [13], have consistently demonstrated that SGLT2is reduce the annual decline in eGFR by approximately 50% in patients with CKD already receiving background ARB therapy. Furthermore, meta-analytical evidence indicates that the renoprotective effects are more pronounced when SGLT2is are initiated at higher baseline eGFR levels, suggesting a greater benefit in earlier CKD stages. This is consistent with the conclusions of a recent review [14] that emphasized that although SGLT2is are effective across all CKD stages, earlier initiation provides more substantial long-term preservation of kidney function.

Predicting the risk of kidney failure is important for identifying individuals who may benefit from early intervention and close monitoring. Several risk prediction models for CKD progression have been developed. The most widely used model is the Kidney Failure Risk Equation (KFRE), which estimates the 2- and 5-year risk of kidney failure using age, sex, eGFR, and albuminuria [15]. Large international collaborations such as the CKD Prognosis Consortium have further characterized the association between kidney measures and adverse outcomes, including kidney failure, cardiovascular disease, and mortality, across diverse populations [16,17]. In addition, disease-specific prediction tools have been developed for specific kidney diseases, such as the International IgA Nephropathy Prediction Tool for IgA nephropathy [18] and the PROPKD Score for autosomal dominant polycystic kidney disease [19]. However, many existing prediction models focus primarily on the short-term risk of kidney failure in patients with established CKD and often require multiple laboratory variables. As a result, simpler approaches that enable the early identification of individuals at high lifetime risk of kidney failure using routinely available measures may complement existing prediction tools.

Given that patients at a higher risk of CKD progression derive greater benefit from timely intervention, early identification of individuals with poor prognosis is essential. This typically requires at least two eGFR measurements over a defined interval to evaluate the trajectory of renal decline. However, an evaluation period of 3–6 months may introduce delays, as limited disease awareness can lead patients to miss follow-up appointments or disengage from outpatient care, thereby losing critical opportunities for effective intervention [20].

Therefore, the decision to initiate RASi and/or SGLT2i therapy should account not only for CKD severity but also for patient age and the likelihood of developing progressive CKD within the individual’s expected lifespan. To address this clinical need, we constructed a prediction model to estimate the lifetime risk of progressive CKD based on a patient’s age and eGFR at a single time point. For this purpose, we utilized data from a large Japanese cohort collected before the widespread use of RASis and SGLT2is [21], thereby allowing an unbiased assessment of the natural course of kidney function decline and enabling risk estimation from a single clinical encounter. These cohort data served as the foundation for the development of our risk prediction model.

## 2. Materials and Methods

### 2.1. Data and Materials

In this study, the mean and standard deviation (SD) of annual eGFR decline were obtained from the dataset used in a previous report [21]. This database consists of annual health checkup records for individuals aged ≥ 40 years from five prefectures in Japan, collected between 1988 and 2003.

As this period predated the widespread use of RASis and SGLT2is, the dataset was intentionally used to approximate the natural history of eGFR decline in the absence of modern renoprotective therapies. Among the individuals in the dataset, 120,727 (39,510 men and 81,217 women) with serum creatinine levels measured twice at an interval of 10 years were included in the original analysis [21]. From this database, sex-specific, age-specific, and baseline eGFR-specific (10 mL/min/1.73 m^2^ bands) means and SDs of the annual eGFR slope were calculated and used as baseline parameters for the present models. In addition, the database provided mean eGFR slopes and SDs classified by the presence or absence of proteinuria. These data were used to model the effects of proteinuria and blood pressure on eGFR decline. The source cohort used for model parameterization is summarized in Supplementary Table S1, including cohort assembly, sex and age distribution, prefecture-specific composition, baseline eGFR categories, and prevalence of proteinuria and hypertension.

### 2.2. Parameters

In our models, we incorporated not only the effects of proteinuria and blood pressure but also additional factors previously reported to influence the eGFR slope. Based on published clinical studies [22,23], we assumed that treatment with RASis would reduce the annual rate of eGFR decline by 30%. Similarly, according to reports on SGLT2is [13,24], we assumed a 50% reduction in the annual rate of eGFR decline during SGLT2i therapy. For smoking status, we referred to previous studies [25,26] and assumed that current smoking increased the annual rate of eGFR decline by 20% compared to nonsmoking values. We acknowledge that the proportional effects assumed for RASis, SGLT2is, and smoking cessation may vary according to baseline eGFR, albuminuria, comorbidities, and clinical course; therefore, these values should be interpreted as simplified modifiers rather than patient-specific causal effects.

### 2.3. Model Structure

This simulation model starts with an individual’s age, sex, and baseline eGFR and estimates how much kidney function is expected to decline over the following year based on the historical average decline observed in people with a similar age and eGFR category. This annual decline is then updated sequentially, year-by-year, using the individual’s current age and eGFR at each step, thereby generating a projected kidney function trajectory over time. Proteinuria and high blood pressure were modeled as factors associated with a faster decline, whereas ARBs and SGLT2is were modeled as factors that reduce the annual decline rate, and current smoking was modeled as a factor that increases it.

In the model, for each individual *i*, aged a  years with an eGFR of g  mL/min/1.73 m^2^, the annual eGFR decline is defined as follows:

$$\Delta_{i}(a,g)=\mu (a,g)+z_{i}\;\sigma (a,g),\;z_{i}∼N(0,1)$$

where $\Delta_{i}(a,g)$ denotes the individual annual decline in eGFR, μ(a,g) is the mean decline, σ(a,g) is the SD of the decline, and $z_{i}$ represents an individual-level random effect. The mean and SD for each age-eGFR combination were modeled as follows:

$$\mu (a,g)=\mu_{\mathrm{base}}(a,g)\times \alpha_{\mathrm{prot}}\times \alpha_{\mathrm{BP}}\times \alpha_{\mathrm{SGLT}2}\times \alpha_{\mathrm{ARB}}\times \alpha_{\mathrm{smoke}}$$

$$\sigma (a,g)=\sigma_{\mathrm{base}}(a,g)\times \beta_{\mathrm{prot}}\times \beta_{\mathrm{BP}}$$

where $\mu_{\mathrm{base}}(a,g)\;$ and $\sigma_{\mathrm{base}}(a,g)\;$ are the sex-, age-, and eGFR-specific baseline mean and SD values of the annual eGFR slope derived from the original database, and the coefficients $\alpha_{*}$ and $\beta_{*}$ are multiplicative factors reflecting the effects of proteinuria, blood pressure category, use of SGLT2is, use of RASis, and smoking status as specified in Parameters Section 2.2. Because the original dataset did not include slope estimates for individuals with eGFR < 30 mL/min/1.73 m^2^, we assumed that individuals with eGFR < 30 mL/min/1.73 m^2^ had the same mean and SD of annual decline as those in the 30–39 mL/min/1.73 m^2^ eGFR stratum. Using this model, we calculated, for each combination of baseline characteristics and risk factors, the probability of reaching a specified eGFR threshold by a given age. This probability was obtained numerically using a bisection algorithm.

### 2.4. Development of the Age + eGFR Heuristic

Age and eGFR were combined into a simple additive heuristic (age + eGFR), motivated by exploratory visualization using age-eGFR heatmaps. These heatmaps estimated the probability of reaching eGFR < 30 or <15 mL/min/1.73 m^2^ by ages 70 and 80 years. Based on the observed risk gradients, age + eGFR values of approximately 115 and 100 were selected as clinically interpretable thresholds representing intensified surveillance and consideration of proactive intervention, respectively.

### 2.5. Calibration and Discrimination

The predictive performance of the age + eGFR heuristic was evaluated using standard calibration and discrimination metrics. Calibration was performed by comparing the predicted risks with the observed event rates across risk strata. Discrimination was evaluated using concordance-based measures to quantify the ability of the heuristic to distinguish between individuals who did and did not experience the outcome.

### 2.6. Decision Curve Analysis

The clinical utility of the age + eGFR heuristic was evaluated using decision curve analysis (DCA), as originally described by Vickers and Elkin [27]. The net benefit was calculated across a range of probability threshold (pt) values (*pt* = 0.05–0.60) using the following standard formulation:

$$\mathrm{Net}\;\mathrm{Benefit}=\frac{TP}{N}-\frac{FP}{N}\times \frac{pt}{1-pt}$$

where TP and FP denote the number of true-positive and false-positive classifications, respectively, and N is the total sample size. Age + eGFR-based thresholds were compared with conventional eGFR-based rules (e.g., eGFR < 60 and <45 mL/min/1.73 m^2^). In this context, the DCA was interpreted as evaluating the clinical usefulness of linking each threshold to different management strategies, ranging from intensified surveillance to consideration of proactive intervention.

### 2.7. Risk Reclassification Analysis

To complement the DCA with patient-level assessments, we performed a hypothetical risk reclassification analysis comparing age + eGFR-based risk categories with conventional eGFR-based stratification. Risk categories were defined a priori as age + eGFR ≥ 115 (low risk), 100–115 (moderate risk), and <100 (high risk), based on clinical interpretability and DCA findings.

The net reclassification improvement (NRI) was calculated according to the framework described by Pencina et al. [28], separately evaluating reclassification among individuals with and without events. Correct reclassification was defined as an upward movement in the risk category among individuals who experienced the outcome and a downward movement among those who did not. Additive and absolute NRI values were reported. Given the clinically motivated nature of the thresholds, the NRI was interpreted as supportive evidence rather than a criterion for threshold optimization.

## 3. Results

### 3.1. Model-Based Probability Estimates for Reaching eGFR Thresholds

**Figure 1:**
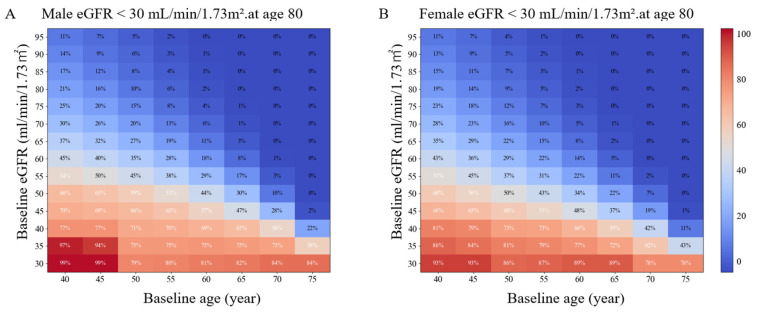
Heatmaps show the estimated probability (%) of reaching eGFR < 30 mL/min/1.73 m^2^. Dark red colors represent higher probabilities of progression. In both sexes, younger baseline age and lower baseline eGFR were associated with substantially higher risks of reaching advanced CKD stages by age 80 years. Panels (**A**,**B**) use sex-specific parameterization estimated separately in males and females from the source dataset. (**A**): Male (**B**): Female. eGFR, estimated glomerular filtration rate; CKD, chronic kidney disease.

**Figure 2:**
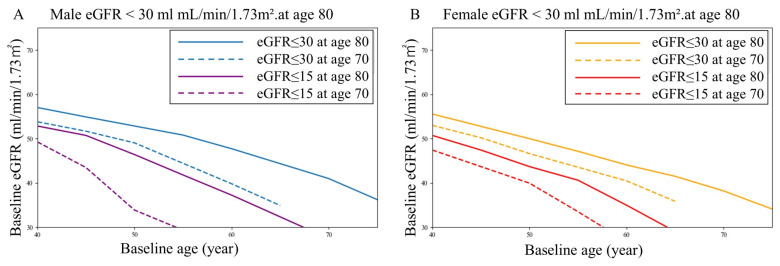
Fifty percent probability borderline of reaching eGFR < 30 mL/min/1.73 m^2^ by age 80 years. Each curve represents the baseline eGFR threshold at a specified age corresponding to a 50% probability of reaching eGFR < 3 0 mL/min/1.73 m^2^ by age 80 years. Lower baseline eGFR and younger baseline age were associated with a higher probability of progression to advanced CKD stages. (**A**): Male, (**B**): Female.

Using age on the horizontal axis and eGFR on the vertical axis, we generated heatmaps of the model-based estimated probability of reaching eGFR < 30 or eGFR < 15 mL/min/1.73 m^2^ by the age of 70 or 80 years (Figure 1, Figures S1–S3). At age 40 years, cases with an eGFR of 57.0/55.6 (Male/Female) mL/min/1.73 m^2^ had a 50% probability of progressing to CKD stage 4 by age 80 years. At age 50, 60, and 70 years, the eGFR values were 52.9/50.0, 47.7/44.1, and 41.0/38.2 mL/min/1.73 m^2^, respectively (Figure 1 and Figure 2).

Similarly, at age 40 years, cases with an eGFR of 52.9/50.7 (Male/Female) mL/min/1.73 m^2^ had a 50% probability of progressing to CKD stage 5 by age 80 years. At age 50, 60, and 70 years, the values were 46.4/43.7, 37.3/35.0, and 27.5/no value mL/min/1.73 m^2^, respectively (Figure 1 and Figure 2). Overall, the eGFR values at the 50% risk boundary for reaching CKD stage 4 or 5 at 80 years of age and CKD stage 4 or 5 at 70 years of age progressively decreased.

### 3.2. Validation Metrics for [Age + eGFR] Heuristic

**Figure 3:**
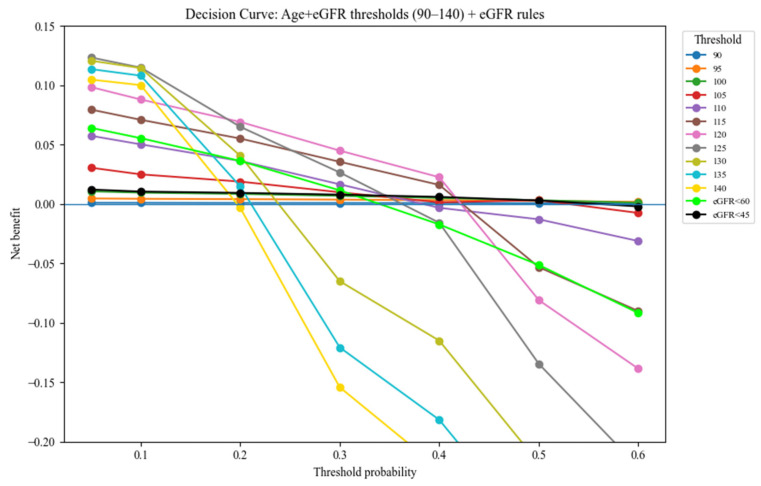
Decision curve analysis for age + eGFR-based thresholds and eGFR-based rules. The net benefit is shown across the threshold probabilities (0.05–0.60). Age + eGFR risk categories (≥120, 100–119, and <100) were defined using clinically motivated thresholds informed by decision curve analysis.

To enable a simple age-adjusted risk estimation, we examined which values of the combined metric age + eGFR were appropriate for predicting prognosis by integrating patient age and baseline eGFR into a single heuristic. DCA was performed to evaluate the clinical utility of age + eGFR-based thresholds across a range of threshold probabilities (Figure 3). Age + eGFR thresholds between 110 and 130 years demonstrated a positive net benefit primarily at lower threshold probabilities, with the magnitude of the net benefit decreasing as the threshold probabilities increased. These thresholds showed a higher net benefit than eGFR-based rules in the low-to-intermediate threshold probability range.

In contrast, age + eGFR = 100 consistently yielded a positive net benefit across the entire range of threshold probabilities examined (pt = 0.05–0.60), a pattern comparable to that observed for eGFR < 45 mL/min/1.73 m^2^. Thresholds of age + eGFR < 100 showed limited or no net benefit at higher pt values. These findings suggest heterogeneity in the clinical implications of age + eGFR thresholds, depending on the assumed decision context. Age + eGFR thresholds of 110–130 demonstrated a positive net benefit mainly at lower pt values, whereas age + eGFR = 100 and eGFR < 45 mL/min/1.73 m^2^ showed a positive net benefit across a wide range of threshold probabilities. Conventional diagnostic performance metrics, including receiver operating characteristic curves (Figure S4), sensitivity, specificity, and Youden index, are provided in Supplementary Table S2 for reference and were not used to define the clinical decision thresholds.

Risk reclassification analysis was performed to compare the age + eGFR-based risk categories with the conventional eGFR-based classification. Using predefined age + eGFR categories (≥115, low risk; 100–114, moderate risk; and <100, high risk), we evaluated patient-level risk reclassification relative to conventional eGFR-based stratification. Among individuals who progressed to eGFR < 30 mL/min/1.73 m^2^ by age 80 years, 6388 were correctly reclassified and 2643 were incorrectly reclassified, resulting in a net positive reclassification of 3745 cases. In contrast, among individuals who did not experience the outcome, 9362 were correctly reclassified and 11,251 were incorrectly reclassified, yielding a net reclassification of −1889 cases. These results indicate that the age + eGFR-based categorization preferentially reclassified a substantial proportion of individuals with events into higher risk categories, while some degree of misclassification occurred among individuals without events. This estimate represents a model-derived approximation based on historical data that has not been externally validated and should not be interpreted as a standalone clinical decision rule (Table 1).

### 3.3. Changes in the Risk of Progression to Reach eGFR < 30 mL/min/1.73 m^2^ by Age 80 Years According to Proteinuria

**Figure 4:**
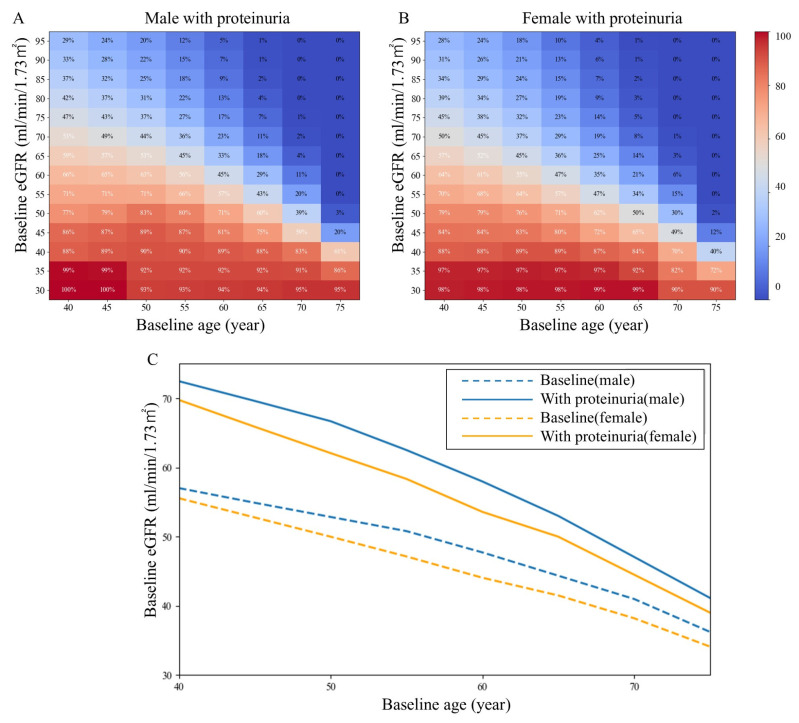
Fifty percent probability borderline of reaching eGFR < 30 mL/min/1.73 m^2^ by age 80 years with and without proteinuria. (**A**,**B**): Heatmaps show the estimated probability (%) of reaching eGFR < 30 mL/min/1.73 m^2^ in populations with/without proteinuria. (**C**): Each curve represents the baseline eGFR threshold corresponding to a 50% probability of reaching eGFR < 30 mL/min/1.73 m^2^ with and without proteinuria for males and females.

A previous report provided the mean eGFR slopes and SDs classified by the presence or absence of proteinuria [21]. A predictive model was constructed considering these factors. Among individuals with proteinuria, the 50% risk threshold was markedly shifted upward, reaching eGFR levels of 12.0–16.7, 10.9–21.5, and 6.4–16.7 at age 40, 50, and 60 years, respectively (Figure 4).

### 3.4. Changes in the Risk of Progression to Reach eGFR < 30 mL/min/1.73 m^2^ by Age 80 Years According to Smoking Status

**Figure 5:**
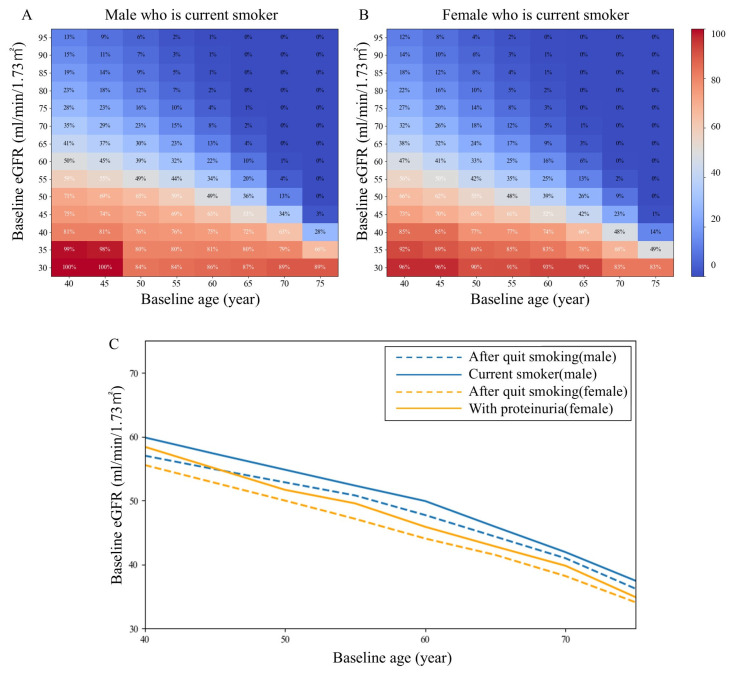
Fifty percent probability borderline of reaching eGFR < 30 mL/min/1.73 m^2^ by age 80 years after smoking cessation. (**A**,**B**): Heatmaps show the estimated probability (%) of reaching eGFR < 30 mL/min/1.73 m^2^ in current smoker. (**C**): Each curve represents the baseline eGFR threshold corresponding to a 50% probability of reaching eGFR < 30 mL/min/1.73 m^2^ for males and females by smoking status.

We then estimated the risk reduction achieved through these interventions. In the smoking status predictive model, smoking was assumed to worsen the rate of eGFR decline by 20%, whereas smoking cessation was assumed to partially attenuate the excess eGFR decline associated with current smoking, approaching the rate observed in nonsmokers. Smoking cessation reduced the probability of progression, a 9.4–11.2% risk reduction (Figure 5). One-way sensitivity analyses varying the assumed effect sizes for smoking cessation (10–30%) showed that the direction and relative magnitude of the model-based risk shifts were preserved (Figure S5).

### 3.5. Changes in the Risk of Progression to Reach eGFR < 30 mL/min/1.73 m^2^ by Age 80 Years After Pharmacological Interventions

**Figure 6:**
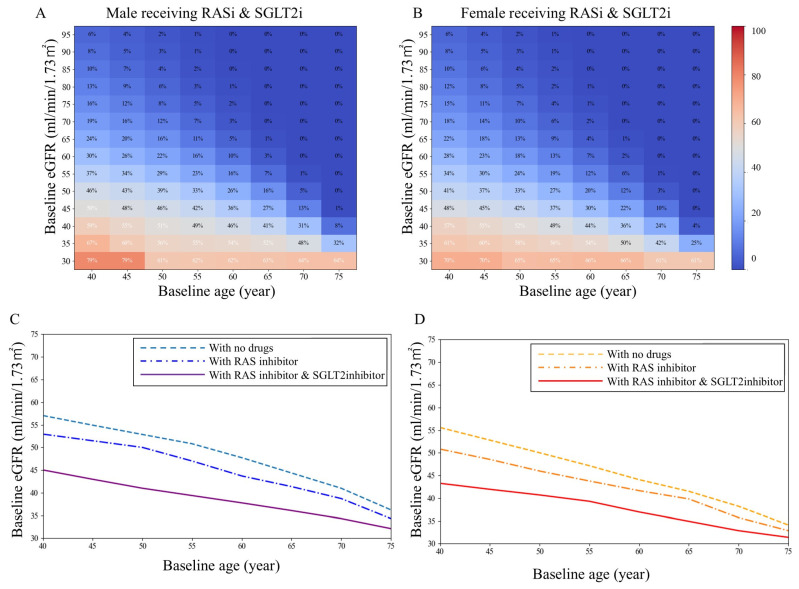
Fifty percent probability borderline of reaching eGFR < 30 mL/min/1.73 m^2^ by age 80 years with pharmacological interventions. (**A**,**B**): Heatmaps show the estimated probability (%) of reaching eGFR < 30 mL/min/1.73 m^2^ with pharmacological intervention (RAS inhibitor [RASi] and/or SGLT2 inhibitor [SGLT2i]). (**C**,**D**): Each curve represents the baseline eGFR threshold corresponding to a 50% probability of reaching eGFR < 30 mL/min/1.73 m^2^ with pharmacological intervention. RAS, renin-angiotensin system; SGLT2, sodium-glucose cotransporter 2.

In the pharmacological intervention predictive model, RASis were assumed to reduce the rate of eGFR decline by 30%, and SGLT2is by 50%, with mutually independent effects. The use of RASis and SGLT2is substantially increased the eGFR thresholds at each age, thereby shifting markedly downward the borderline for progression to eGFR < 30 mL/min/1.73 m^2^ by age 80 years (Figure 6, Figures S7 and S8). As a result, pharmacological intervention reduced the probability of progression by 31.2–40.0% in the model-based risk reduction.

In 40-year-old men, pharmacological interventions were associated with a reduction in estimated probability of reaching eGFR < 30 mL/min/1.73 m^2^ by age 80 years across all patient groups represented by each assumed probability distribution (30.0%→17.9%, 40.0%→24.4%, 50.0%→32.1%, 60.0%→40.2%, and 70.0%→47.8%). One-way sensitivity analyses varying the assumed effect sizes for RASis (20–40%) and SGLT2is (30–60%) showed that the direction and relative magnitude of the model-based risk shifts were preserved across scenarios (Figures S7 and S8). Larger assumed effects yielded greater shifts in the probability boundaries, whereas smaller effects produced attenuated but directionally consistent changes, supporting the qualitative robustness of the primary findings.

Intervention analyses represent model-based scenario projections rather than empirically observed treatment effects in the source cohort. Literature-based effect sizes for each intervention were applied as constant, mutually independent proportional modifiers of the annual eGFR decline rate without modeling interactions or time-varying efficacies. Accordingly, these scenarios implicitly assume immediate and sustained effects across ages and baseline eGFR levels, which may overestimate the benefits in some clinical settings (e.g., in patients without diabetes or with low levels of proteinuria).

## 4. Discussion

CKD is highly prevalent worldwide, affecting more than 10% of the global adult population. Recent global estimates suggest that more than 800 million individuals have CKD [29]. Despite this large burden, CKD remains underdiagnosed and undertreated across healthcare systems worldwide, including in high-income countries [2,30]. The 2024 Kidney Disease: Improving Global Outcomes (KDIGO) guideline [3] classifies CKD risk based on eGFR and albuminuria categories, and these classifications provide thresholds for referral to nephrology specialists. However, the current guidelines offer limited guidance on which patients should receive early and intensive pharmacological interventions. We selected eGFR < 30 mL/min/1.73 m^2^ as the primary endpoint because this threshold represents a clinically meaningful transition to CKD stage G4, at which the risk of progression to kidney failure increases substantially and referral to specialized nephrology care is recommended, particularly in older adults.

Using age + eGFR thresholds of 115 and 100, we proposed a simple age-adjusted risk stratification model that classifies individuals into low-, moderate-, and high-risk categories. An age + eGFR threshold of 115 provided a positive net benefit, primarily at lower threshold probabilities, supporting its use as a marker for intensified surveillance with a minimal burden of false-positive individuals. In contrast, an age + eGFR threshold of 100 consistently showed a positive net benefit across a broader range of threshold probabilities, comparable to the conventional eGFR < 45 mL/min/1.73 m^2^ criterion, suggesting its relevance as a boundary for considering more proactive interventions.

Risk reclassification analysis was conducted as a complementary patient-level assessment to evaluate how these DCA-informed thresholds redistributed individuals across clinically defined risk categories. While conventional reclassification metrics showed limited incremental improvement when comparing age + eGFR = 100 with eGFR < 45 mL/min/1.73 m^2^, examination of the reclassified individuals revealed that many were younger patients with eGFR values ≥ 45 mL/min/1.73 m^2^ who were nonetheless identified as high risk by the age + eGFR approach. Clinically, this subgroup represents individuals in whom earlier intervention and intensified management are likely to be particularly effective, despite not meeting the traditional eGFR-based criteria. Accordingly, the clinical benefit of age + eGFR-based stratification may extend beyond that captured by reclassification statistics alone, underscoring the importance of interpreting reclassification analyses within appropriate clinical contexts.

Previous studies have demonstrated that the risks of progressive CKD, all-cause mortality, cardiovascular mortality, and all-cause hospitalization begin to increase at eGFR levels below 60 mL/min/1.73 m^2^ [31]. This underscores the importance of preventing transition to CKD stage 3. Although several effective therapeutic options, including lifestyle modification, RASis, and SGLT2is, are available to delay CKD progression, the real-world challenge is to identify individuals at high risk before symptoms arise. Current CKD classification systems do not incorporate age, which limits their ability to precisely stratify risk among patients with mild reductions in eGFR.

Furthermore, in routine clinical practice, healthy adults usually undergo eGFR testing no more than once a year during health checkups. Even when abnormalities are detected, some individuals do not undergo medical consultation. Risk stratification methods based on eGFR decline require at least two measurements over a defined interval to evaluate the trajectory of renal decline. For example, patients classified as having CKD G3a without proteinuria or hematuria are often reevaluated one year later. However, our results show that individuals in their 40s with eGFR in the G3a range (45–60 mL/min/1.73 m^2^) have an approximately 50% probability of developing progressive CKD by age 80 years, indicating that reliance on repeated measurements may delay necessary intervention in a substantial proportion of patients at high risk.

Another important point is that, in contemporary clinical practice, patients with progressive CKD are usually treated with RASis, including ACEIs and ARBs, and are commonly advised regarding salt restriction, exercise, and other lifestyle modifications. As a result, the natural course of untreated CKD is increasingly difficult to observe directly in modern cohorts. In this context, the historical cohort used in the present study may retain value because it captures renal trajectories from an era before widespread implementation of these interventions and therefore provides a useful approximation of the underlying untreated risk. From a clinical perspective, such information may support risk communication with patients and may help clinicians make initial decisions regarding follow-up intensity and the need for early therapeutic intervention. At the same time, this advantage should be balanced against the limited direct applicability of historical data to current treated populations.

Adherence is also a major challenge when initiating lifestyle or pharmacological interventions in individuals who are asymptomatic. Therefore, it is essential to provide patients with quantitative estimates of their absolute risk and the magnitude of risk reduction achievable through treatment. Our findings demonstrate that the risk-lowering effects of smoking cessation and RAS and SGLT2 inhibition can be visualized and communicated directly, which may improve patient understanding and adherence.

Previous studies have indicated associations between blood pressure and the rate of eGFR decline [32,33]. In our analysis, we did not observe meaningful differences in progression probabilities across the blood pressure categories. This may be because the original dataset classified blood pressure into only broad groups using mean values of 96 and 106 mmHg, which may have limited the ability to detect more nuanced effects. Moreover, because our data were derived from health checkups, information on antihypertensive medication use was unavailable. Thus, the observed blood pressure values may reflect treatment effects rather than the underlying pathophysiology.

Our model has several limitations. First, we did not incorporate biphasic eGFR trajectories (initial dip followed by a slower decline) that could occur with RASi and SGLT2i treatment. Second, literature-based intervention effects were implemented as constant proportional modifiers without time-varying efficacies or interactions, although the true effects varied according to albuminuria, comorbidities, and underlying kidney disease. Moreover, the health checkup dataset lacked etiologic/phenotypic information, and we could not model cause-specific trajectories or assess whether the proposed heuristic could be generalized across CKD subtypes. Third, because data were limited for eGFR < 30 mL/min/1.73 m^2^, we approximated the decline parameters using the 30–39 mL/min/1.73 m^2^ stratum; as progression may accelerate and become more heterogeneous in advanced CKD, late-stage projections should be interpreted with caution. Fourth, the model was parameterized from cross-sectional summary statistics rather than longitudinal individual-level data and thus cannot fully capture within-person variability, time-varying risk factors, or nonlinear trajectories. Finally, heterogeneity in annual eGFR decline was modeled with a normal random effect, which may underrepresent skewed distributions and extreme declines (e.g., acute kidney injury-related drops). Alternative distributional structures warrant future validation in longer-term, well-characterized cohorts. In addition, external validation in independent and contemporary cohorts will be essential to determine whether the proposed framework is generalizable across different patient populations and healthcare settings, particularly given changes in CKD management and population characteristics over time.

## 5. Conclusions

Our results indicate that, within the constraints of a simulation model parameterized using historical aggregated data, the combination of age and eGFR may provide an interpretable framework for the initial risk stratification of long-term decline in kidney function. Age + eGFR thresholds of 115 and 100, derived from a single-point assessment, should be viewed as illustrative markers for risk stratification rather than as thresholds for definitive clinical decision-making. Future studies using individual-level longitudinal data are needed to evaluate whether this approach improves clinical decision-making and patient outcomes.

## Figures and Tables

**Table 1:** Hypothetical Risk Reclassification Analysis.

(mL/min/1.73 m^2^)		Age + eGFR		
		≧115	<115	<100
		Low Risk	Moderate Risk	High Risk
eGFR < 30 at age 80				
eGFR ≧ 60	Low Risk	8330	5375	0
eGFR < 60	Moderate Risk	1568	4698	1013
eGFR < 45	High Risk	0	1075	317
eGFR ≧ 30 at age 80				
eGFR ≧ 60	Low Risk	71,159	10,510	0
eGFR < 60	Moderate Risk	8468	6746	741
eGFR < 45	High Risk	0	894	94
	Case with events (n)		Case without events (n)	
Correct reclassification	6388		9362	
Incorrect reclassification	2643		11,251	
Net reclassification	3745		−1889	
Additive NRI	14.82%			
Absolute NRI	1.53%			

Reclassification was assessed relative to eGFR-based categories. NRI values reflect a sensitivity-prioritized strategy and should be interpreted alongside the decision curve analysis. NRI, net reclassification improvement.

## Data Availability

The original contributions presented in this study are included in the article/Supplementary Material. Further inquiries can be directed to the corresponding authors.

## Supplementary Materials

The following supporting information can be downloaded at: https://www.mdpi.com/article/10.3390/jcm15072653/s1.
