# Supplementary material for: Predicting Lifetime Risk of Kidney Failure Using Age and a Single eGFR Measurement
Source: J Clin Med. 2026 Mar 31;15(7):2653. doi: 10.3390/jcm15072653 (PMC13073389; doi:10.3390/jcm15072653)
Supplement: Supplementary file 1 [file jcm-15-02653-s001.zip › jcm-4084325-supplementary.pdf]

Supplementary Materials

Predicting Lifetime Risk of Kidney Failure Using Age and a Single eGFR Measurement

Ryo Enoki [1,*], Mariko Miyazaki [1], Enyu Imai [2], Tetsuhiro Tanaka [1] and Koji Okamoto [1,*]

Table S1. Published Aggregated Characteristics of the Historical Japanese Health-Checkup Cohort Used for Model Parameterization

Section	Variable	Value
Cohort assembly	Source population	290,268 individuals aged ≥40 years from annual health examination programs in five prefectures of Japan
	Sex in source population	107,145 men; 183,123 women
	Calendar period of source examinations	Earlier examinations: 1988–1993; later examinations: 1998–2003
	Participants included in original longitudinal analysis	120,727 participants with serum creatinine measured twice at an interval of approximately 10 years
	Inclusion fraction	41% of the original source population
	Sex in analytic cohort	39,510 men; 81,217 women
Available measurements	Urinary protein measured	117,865 participants
	Proteinuria	2,054/117,865 (1.7%); defined as dipstick urinary protein ≥1+
	Blood pressure measured	120,727 participants
	Hypertension	16,722/120,727 (13.9%); defined as mean blood pressure ≥106 mmHg
Age distribution in analytic cohort	40–49 years	30,864 (25.6%)
	50–59 years	37,848 (31.4%)
	60–69 years	42,569 (35.3%)
	70–79 years	9,446 (7.8%)
Sex-specific age distribution	Men aged 40–49 / 50–59 / 60–69 / 70–79 years	9,331 / 10,555 / 15,823 / 3,801
	Women aged 40–49 / 50–59 / 60–69 / 70–79 years	21,533 / 27,293 / 26,746 / 5,645

Section	Variable	Value
Baseline kidney function distribution	Initial eGFR >70 mL/min/1.73 m²	42.72%
	Initial eGFR 60–69 mL/min/1.73 m²	35.91%
	Initial eGFR 50–59 mL/min/1.73 m²	17.80%
	Initial eGFR 40–49 mL/min/1.73 m²	3.29%
	Initial eGFR 30–39 mL/min/1.73 m²	0.26%
	Initial eGFR <30 mL/min/1.73 m²	0.01%
Prefecture-specific composition	Okinawa	29,673 participants (11,324 men; 18,349 women); comparison years 1993 and 2003
	Ibaraki	85,985 participants (25,262 men; 60,723 women); comparison years 1993 and 2003
	Hokkaido	967 participants (395 men; 572 women); comparison years 1991 and 2001
	Tokyo	2,550 participants (1,928 men; 622 women); comparison years 1992 and 2001
	Fukuoka	1,556 participants (605 men; 951 women); comparison years 1988 and 1998

Abbreviations: Data were extracted from the published source article and represent aggregated summary statistics only; no individual-level participant data were available for the present study.

Percentages for age distribution were calculated from reported counts in the original article. Proteinuria was defined as dipstick urinary protein ≥1+, and hypertension was defined as mean blood pressure ≥106 mmHg.

More detailed individual-level baseline variables, cross-tabulated subgroup characteristics, and additional exclusion criteria beyond repeat serum creatinine availability were not available from the published source report.

Figure S1. Heatmap of the probability of having eGFR < 15 mL/min/1.73m² at age 80

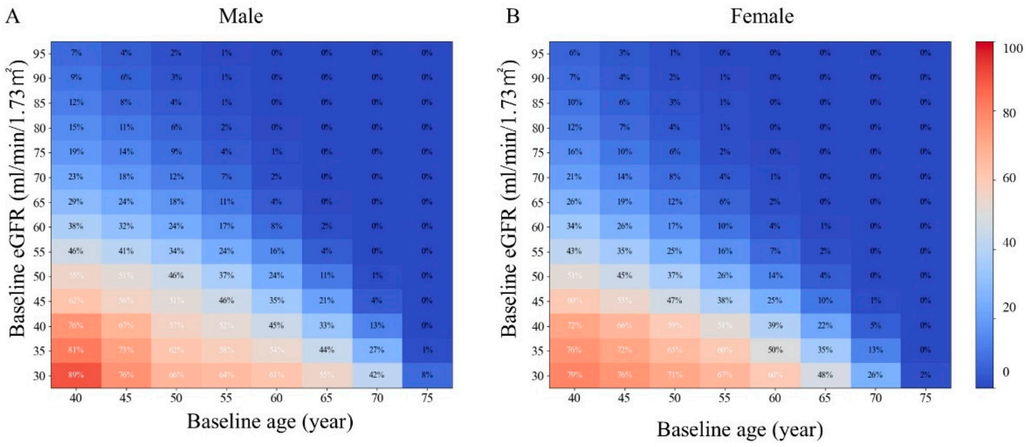

Figure S2. Heatmap of the probability of having eGFR < 30 mL/min/1.73 m² at age 70

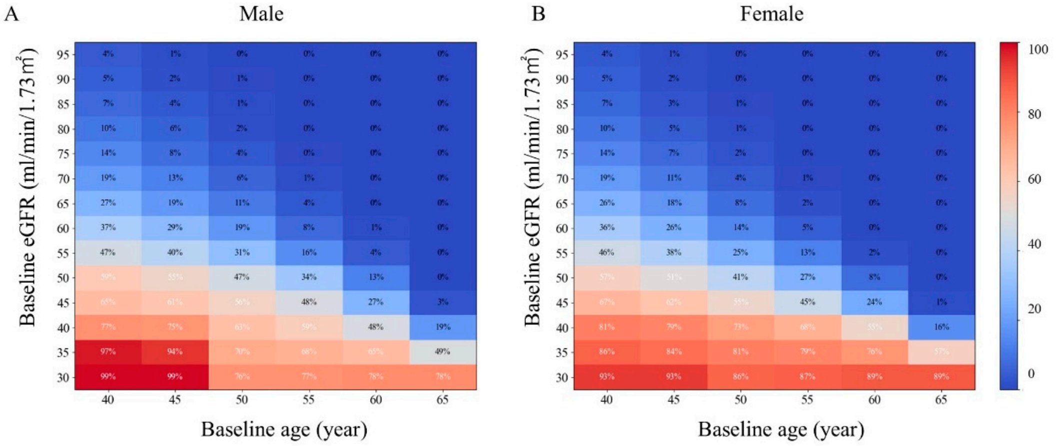

FigureS3. Heatmap of the probability of having eGFR < 15 mL/min/1.73 m² at age 70

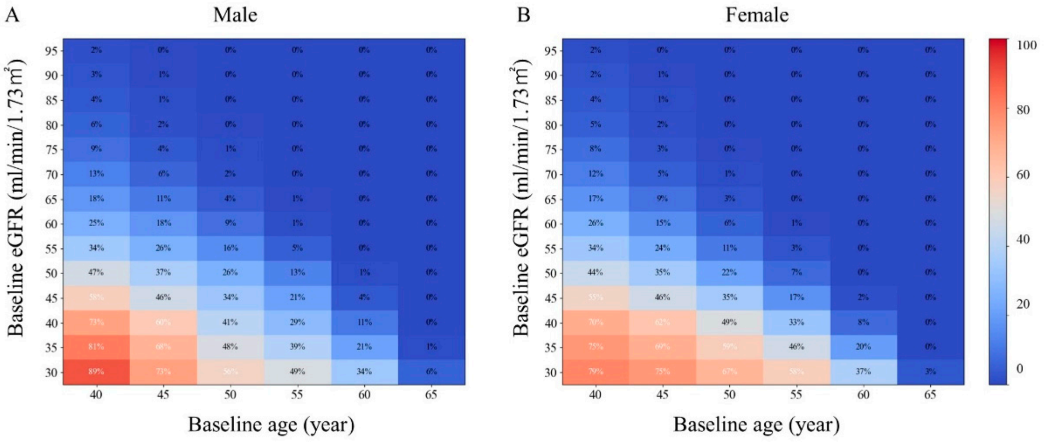

Figure S4. ROC curve for rule: predict positive if (Age + eGFR) < c

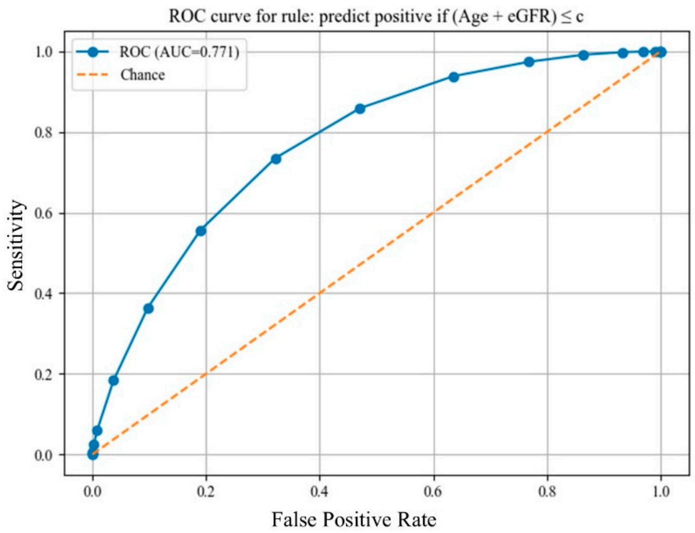

c=100: TPR=0.060, FPR=0.008, Specificity=0.992

c=110: TPR=0.364, FPR=0.098, Specificity=0.902

c=115: TPR=0.557, FPR=0.190, Specificity=0.810

c=120: TPR=0.735, FPR=0.322, Specificity=0.678

Supplementary Table S2. Stats of predictive thresholds

Criteria		Sensitivity	Specificity	PPV	NPV	Diagnostic OR	Youden index
Age + eGFR	80	0.000	1.000	0.932	0.815	60.6	0.000
	90	0.005	1.000	0.784	0.816	16.1	0.005
	100	0.060	0.992	**0.614**	0.823	7.4	0.051
	110	0.364	0.902	0.459	0.862	**5.3**	0.266
	120	0.735	0.678	0.341	0.919	5.8	**0.413**
	130	0.938	0.365	0.251	0.963	8.7	0.303
	140	0.992	0.135	0.207	0.986	19.0	0.127
	150	1.000	0.030	0.190	0.998	106.8	0.030
	160	1.000	0.003	0.186	1.000	NA	0.003
eGFR	< 60	0.386	0.830	0.341	0.856	3.1	0.217
	< 45	0.062	0.990	0.585	0.823	6.5	0.052

Conventional diagnostic performance metrics are shown for Age + eGFR thresholds and eGFR-based criteria. These measures are provided for reference only and were not used to define clinical decision thresholds.

Figure S5. One-way sensitivity analyses (Smoking)

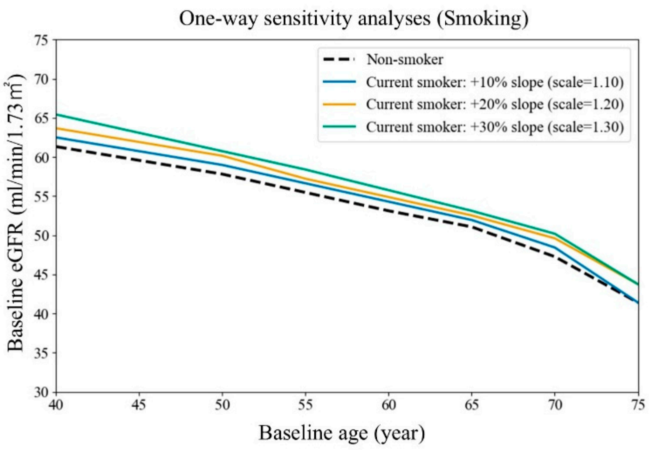

One-way sensitivity analyses illustrating the impact of varying assumed proportional effect sizes for clinical interventions on model-based probability boundaries for reaching eGFR <30 mL/min/1.73 m² by age 80.

The assumed effect of smoking cessation was varied from a 10% to 30% reduction in annual eGFR decline (base case: 20%), the effect of renin–angiotensin system inhibitors (RASi) from 20% to 40% reduction (base case: 30%), and the effect of sodium–glucose cotransporter 2 inhibitors (SGLT2i) from 30% to 50% reduction (base case: 50%).

Figure S6. Heatmap of the probability of having eGFR < 30 mL/min/1.73 m² at age 80 among with pharmacological intervention.

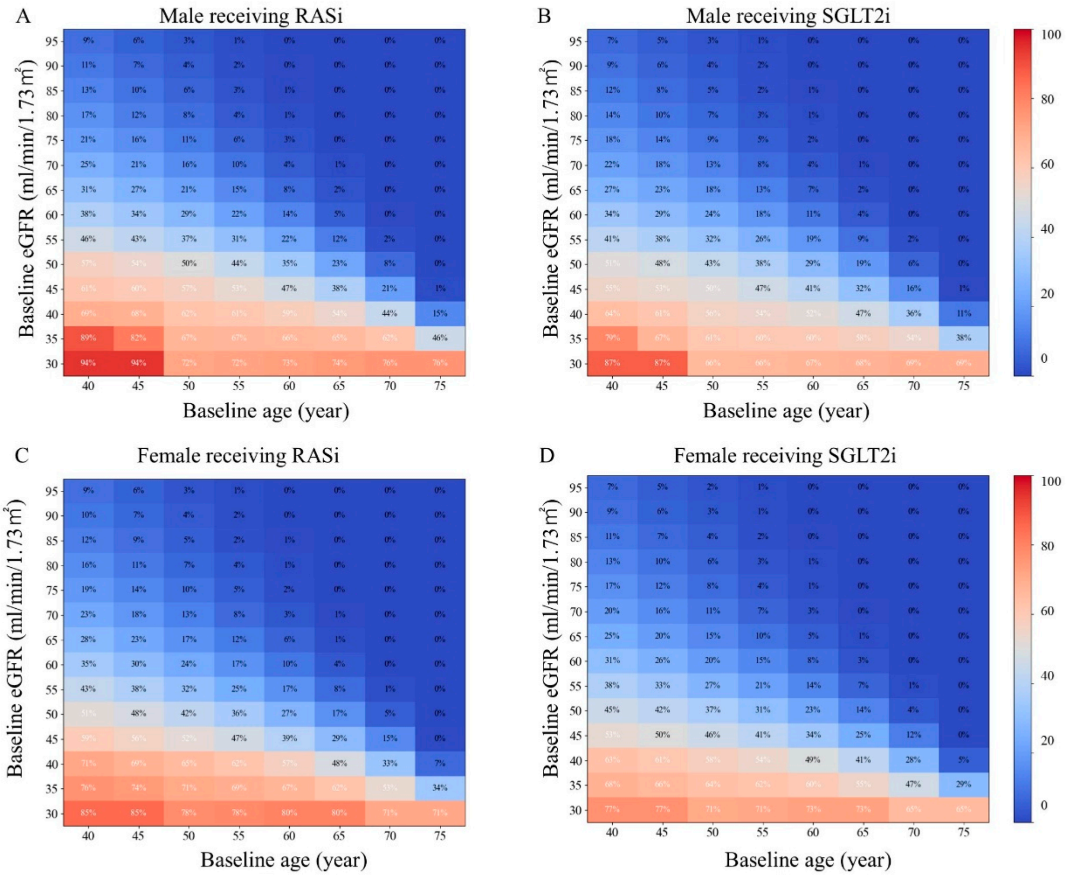

Figure S7. One-way sensitivity analyses (RASi)

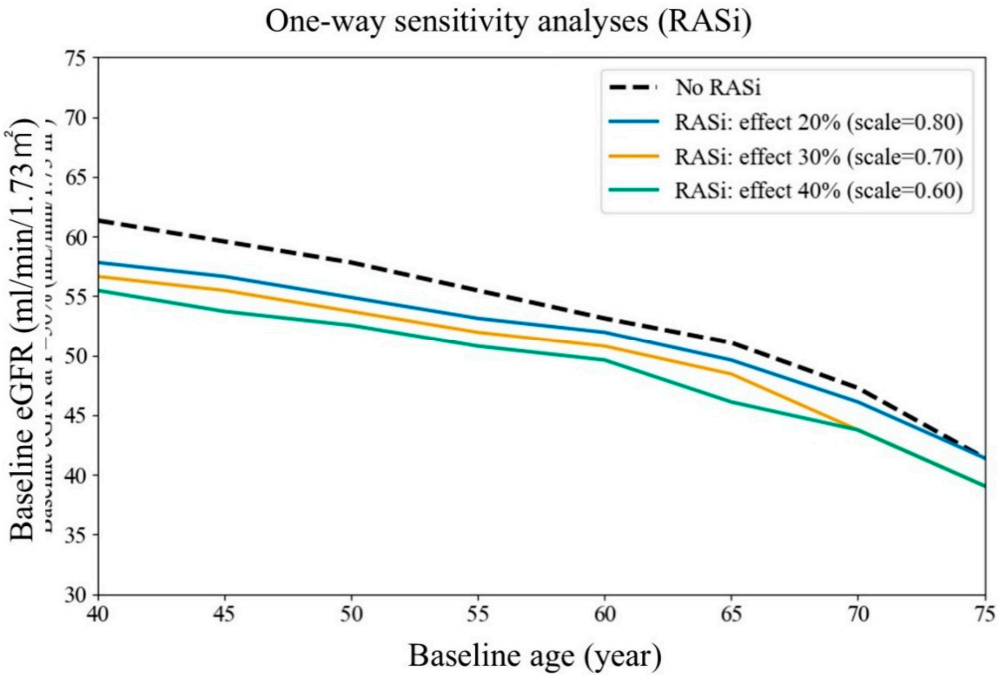

One-way sensitivity analyses illustrating the impact of varying assumed proportional effect sizes for clinical interventions on model-based probability boundaries for reaching eGFR <30 mL/min/1.73 m² by age 80.

The assumed effect of RASi was varied from a 20% to 40% reduction in annual eGFR decline (base case: 30%),.

Figure S8. One-way sensitivity analyses (SGLT2i)

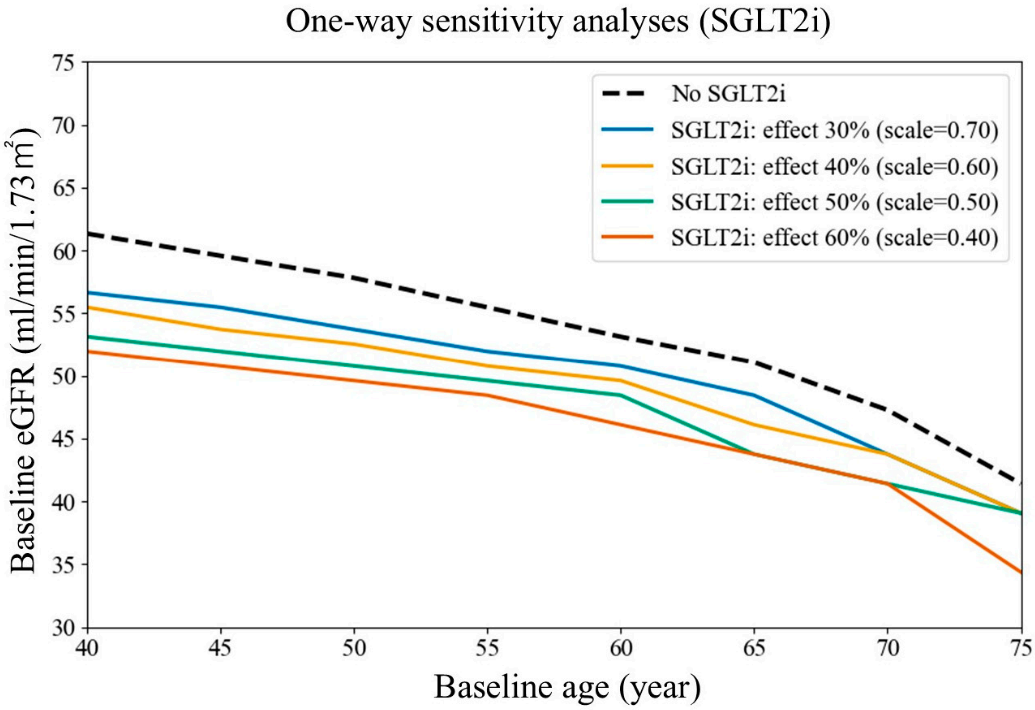

One-way sensitivity analyses illustrating the impact of varying assumed proportional effect sizes for clinical interventions on model-based probability boundaries for reaching eGFR <30 mL/min/1.73 m^2 by age 80.

The assumed effect of SGLT2i was varied from a 30% to 60% reduction in annual eGFR decline (base case: 50%),
